# Expansion of the Genotypic and Phenotypic Spectrum of *TCTN3*-Related Joubert Syndrome

**DOI:** 10.3390/genes16060706

**Published:** 2025-06-13

**Authors:** Mariangela Lo Giudice, Eugenia Borgione, Marika Giuliano, Sandro Santa Paola, Francesco Domenico Di Blasi, Rosa Pettinato, Corrado Romano, Carmela Scuderi

**Affiliations:** 1 Oasi Research Institute—IRCCS, Via Conte Ruggero 73, 94018 Troina, Italy; mlogiudice@oasi.en.it (M.L.G.); eborgione@oasi.en.it (E.B.); mgiuliano@oasi.en.it (M.G.); ssantapaola@oasi.en.it (S.S.P.); fdiblasi@oasi.en.it (F.D.D.B.); rpettinato@oasi.en.it (R.P.); cscuderi@oasi.en.it (C.S.); 2 Section of Clinical Biochemistry and Medical Genetics, Department of Biomedical and Biotechnological Sciences, University of Catania, 95123 Catania, Italy

**Keywords:** *TCTN3*, Joubert syndrome, thickened corpus callosum

## Abstract

Background/Objectives: Joubert syndrome (JS, MIM 213300) is a rare genetic condition characterized by respiratory control disturbances, abnormal eye movements, ataxia, cognitive impairment, and the notable agenesis of the cerebellar vermis. The molar tooth sign visible in magnetic resonance imaging of the brain serves as a diagnostic tool for JS. Variants in the *TCTN3* gene can lead to the development of several diseases, including JS type 18, Orofaciodigital syndrome IV, and Meckel–Gruber syndrome. Methods: We performed whole-exome sequencing (WES) in a 49-year-old woman with JS characterized by severe intellectual disability, ataxic gait, agenesis of the cerebellar vermis leading to the molar tooth sign, dystonic movements, strabismus, and nystagmus. Moreover, the patient also showed a thickened corpus callosum. Results: Molecular analysis through WES revealed the heterozygous variants c.182dup (p.G62Wfs*18) and c.1452+4del in the *TCTN3* gene, expanding our understanding of the genetic diversity and potential phenotypic implications associated with *TCTN3* variations. Conclusions: To our knowledge, this is the first patient with JS and a thickened corpus callosum. Moreover, a thickened corpus callosum has never been identified in patients with pathogenic variants of the *TCTN3* gene.

## 1. Introduction

Joubert syndrome (JS, MIM 213300) is a rare clinical and genetic heterogeneous condition characterized by respiratory control disturbances, abnormal eye movements, ataxia, cognitive impairment, and the notable agenesis of the cerebellar vermis. The “molar tooth sign” (MTS), visible on magnetic resonance imaging (MRI) of the brain, serves as a diagnostic hallmark for JS and is characterized by thickened and horizontally oriented superior cerebellar peduncles, along with a deep interpeduncular fossa [1,2]. However, the MTS is also present in many other disorders, collectively known as Joubert-related disorders, such as Dekaban–Arima, COACH, Senior–Løken, and Orofaciodigital IV syndromes [3]. Apart from the MTS, additional brain anomalies have been described in individuals with JS, including occipital encephalocele, corpus callosum agenesis and dysgenesis, ventriculomegaly, neural migration defects, hypothalamic hamartoma, and abnormal folial organization of the cerebellar hemispheres [4,5]. Psychomotor milestone acquisition is almost always delayed, and many patients present with intellectual disability (ID) [6]. Cognitive abilities vary widely, ranging from normal intelligence quotient to severe impairments in motor, language, and adaptive functioning. Cognitive deficits are often associated with behavioral and autism spectrum disorders, as well as sleep disturbances [7]. In addition to central nervous system (CNS) features, some individuals with JS may exhibit ocular issues (such as chorioretinal coloboma and progressive retinal dystrophy), kidney problems (nephronophthisis), liver abnormalities (including ductal plate malformation and fibrosis), and/or skeletal anomalies (dystrophy and polydactyly) [4]. JS shares a genetic and phenotypic overlap with the more severe Meckel–Gruber Syndrome (MKS), which is often characterized by the co-occurrence of occipital encephalocele, cystic–dysplastic kidney disease, liver fibrosis, and perinatal lethality [8]. JS is classified among ciliopathies, a group of genetic disorders caused by defects in the structure or function of cilia—microtubule-based organelles projecting from the surface of most differentiated cells. These cilia play essential roles in various cellular processes, acting as environmental sensors that transduce sensory, chemical, or mechanical signals. Moreover, cilia are involved in critical signaling pathways during development and homeostasis [9]. Defects in the primary cilium can lead to a wide range of clinical phenotypes, affecting nearly every major body system, including the brain, eyes, liver, kidneys, skeleton, and limbs [10].

Based on the clinical features observed in patients, JS is classified into several subtypes [11,12].

In the classical or purely neurological JS subtype, the most frequently observed neurological symptoms include irregular breathing rhythms, rapid respiration, obstructive sleep apnea, hypotonia, abnormal eye and tongue movements, cognitive impairment, and coordination problems such as ataxia. The JS subtype with ocular involvement commonly presents with atypical retinal development, coloboma, involuntary eye movements (nystagmus), misalignment of the eyes (strabismus), and ptosis. JS with renal involvement typically includes isolated nephronophthisis, a combination of nephronophthisis and cystic dysplasia, or presentations resembling autosomal recessive polycystic kidney disease. Forms of JS with both ocular and renal involvement are referred to as Senior–Løken Syndrome. JS with hepatic involvement (also known as JS Type 7/Meckel Syndrome Type 5/COACH Syndrome) is characterized by congenital hepatic fibrosis and may present with portal hypertension, elevated liver enzymes, cholangitis, gastroesophageal varices, and thrombocytopenia.

A less common subtype is JS with oral–facial–digital features, which may include craniofacial abnormalities such as cleft lip and/or palate, a midline groove of the tongue, hamartomas of the tongue or gums, hypertelorism, micrognathia, and polydactyly, often postaxial and typically affecting both hands and feet. Another subtype is characterized by abnormalities of the corpus callosum. Finally, JS with Jeune asphyxiating thoracic dystrophy features skeletal dysplasia, including a narrow thoracic cage, short ribs, shortened tubular bones, a “trident-shaped” acetabular roof, rhizomelic limb shortening, cone-shaped epiphyses, brachydactyly, and often polydactyly.

JS predominantly shows autosomal recessive inheritance caused by biallelic variants in about 40 known genes. Exceptions include mutations in the X-linked *OFD1* gene [13] and heterozygous variants in the ZNF423 gene that lead to autosomal dominant inheritance [14]. The prevalence of JS at birth is estimated at approximately 1.7:100,000 live births [15].

The human tectonic *TCTN3* gene spans 30.7 kb, is mapped to chromosome 10q24.1, consists of 14 exons, and encodes TCTN3, a 607-amino acid membrane protein localized to the primary cilium. TCTN3 is essential for activation of the Sonic Hedgehog (Shh) signaling pathway and neural tube development [16,17]. Mutations in *TCTN3* can cause several diseases, including JS type 18 (OMIM 614815), Orofaciodigital syndrome IV (OFD type IV, OMIM 258860), and Meckel–Gruber Syndrome (MKS, OMIM 249000)**.**

To date, only five variants (three missense, one splicing, and one frameshift) in the *TCTN3* gene have been identified as causative of JS [4,18,19,20,21]. In this report, we present a European proband with the MTS, thickened corpus callosum, ataxia, and ID. Molecular analysis through whole-exome sequencing (WES) revealed the heterozygous variants c.182dup (p.G62Wfs*18) and c.1452+4del in the *TCTN3* gene. This case expands the clinical phenotype and genotype spectrum of TCTN3-associated diseases.

## 2. Materials and Methods

### 2.1. Patient Examination

The patient was referred to our institute for a comprehensive diagnostic assessment to further investigate the underlying condition. A thorough clinical and instrumental evaluation was performed, including the assessment of adaptive and cognitive profiles, neurological examination, neuroradiological imaging (brain MRI), neurophysiological studies (EEG), and medical imaging (ultrasound examination of the abdominal cavity).

### 2.2. Next-Generation Sequencing Analysis

After obtaining informed consent, blood samples were collected from the patient and her parents, and genomic DNA was extracted following standard protocols. To investigate the genetic basis of the disease, whole-exome sequencing (WES) was performed on the patient’s DNA. Library preparation was carried out using the Nextera Flex for Enrichment Sample Prep kit (Illumina, San Diego, CA, USA) according to the manufacturer’s instructions. Sequencing was conducted on an Illumina NextSeq500 platform using a 2 × 150 bp paired-end reads protocol. Raw sequencing reads were aligned to the human reference genome (GRCh37/hg19), and binary alignment map (BAM) files were visualized using the Integrative Genomics Viewer (IGV) software (v2.5.3). Variant calling was performed using the Genome Analysis Toolkit (GATK) pipeline (https://gatk.broadinstitute.org/hc/en-us), and variants were annotated with ANNOVAR (http://wannovar.wglab.org). Variants classified as benign were excluded from further analysis. Remaining variants were prioritized and classified based on potential clinical significance into pathogenic, likely pathogenic, or variants of uncertain significance (VUS), according to the following criteria: (i) nonsense or frameshift variants in genes known to cause disease via haploinsufficiency or loss-of-function mechanisms; (ii) missense variants located within critical or functional domains; (iii) variants affecting canonical splice sites; and (iv) variants classified or predicted as pathogenic or deleterious in ClinVar. In alignment with the patient’s pedigree and phenotype, priority was given to rare variants (allele frequency <1% in public databases, including the 1000 Genomes Project [http://1000genomes.org], Exome Aggregation Consortium (ExAC, http://exac.broadinstitute.org), and Genome Aggregation Database (gnomAD, https://gnomad.broadinstitute.org/ containing 125,748 exomes), that fit a recessive inheritance model (e.g., compound heterozygous or homozygous) or a de novo model. Deleterious single nucleotide variants were predicted using MutationTaster (http://www.mutationtaster.org/, accessed on 1 October 2024), SpliceRover (http://bioit2.irc.ugent.be/rover/splicerover, accessed on 1 October 2024), and SpliceAI (https://spliceailookup.broadinstitute.org/, accessed on 10 June 2025).

### 2.3. Sanger Sequencing

PCR primers for exons 1 and 12 of the *TCTN3* gene were designed using the software Vector NTI Advance 10.3.0 (Informax, Frederick, MD, USA) to verify the variants identified by Sanger sequencing. The reference sequences used were the *TCTN3* gene, (NM_015631.6) and the TCTN3 protein (NP_056446.4). PCR reactions were performed according to the manufacturer’s instructions. Briefly, 50 μL reaction volumes containing 200 ng genomic DNA, 1X PCR reaction buffer, 0.2 mM of each dNTP, 1 μM of each primer, and TaqDNA polymerase (Roche, Mannheim, Germany) were used. PCR cycling conditions used for the amplification included an initial denaturation step at 94 °C for 5 min, followed by 35 cycles of 30 s at 94 °C, 45 s at 58 °C, 40 s at 72 °C and a final extension step at 72 °C for 7 min.

The sequencing of PCR products was performed using BigDye Terminator chemistry (Thermo Fisher Scientific, Vilnius, Lithuania) in both forward and reverse directions on an ABI 3130 automated sequencer (Applied Biosystems, Foster City, CA, USA). Patient sequence data were aligned and compared with the corresponding wild-type reference sequence.

### 2.4. cDNA Analysis

RNA extraction from peripheral blood leucocytes was performed using the RNeasy Mini Kit (Qiagen, Hilden, Germany), following the manufacturer’s protocol. RNA quality and quantity were assessed by spectrophotometry. To prevent genomic DNA contamination during PCR, samples were briefly incubated at 42 °C with a specific Wipeout buffer of the QuantiTect Reverse Transcription Kit (Qiagen, Hilden, Germany). Reverse transcription of 500 ng of total RNA from sample was then carried out in a final volume of 20 μL to generate cDNA for sequencing. PCR amplification was performed on the cDNA using primers designed on exon 10 and exon 13 of *TCTN3* (forward primer: 5′-CTCAATGACCCTCTTACAGAGCC-3′; reverse primer: 5′-TGGCACTGGTATAGGAATCGAA-3′). The resulting PCR products were analyzed by electrophoresis on a 1% agarose gel. All reactions were conducted according to the manufacturer’s instructions.

## 3. Results

### 3.1. Patient’s Clinical History

The patient, a 49-year-old woman, was born following a spontaneous full-term delivery. At birth, she presented with a weak cry and cyanotic asphyxia. During the first two months of life, she was hospitalized in the pediatric department, where clonic contractions of the diaphragm followed by cyanosis were reported. Psychomotor development was significantly delayed: she began walking at 11 years of age, her language was limited to 6–7 words, and sphincter control was never achieved.

At the time of assessment, the patient exhibited severe intellectual disability. The phenotype was characterized by brachycephaly, relative macrocephaly, an elongated and asymmetric face with the right side more affected than the left, flat occiput, narrow forehead, prominent orbital ridges, large ears with hypoplastic lobes, prominent nose, thin upper lip, eversion of the lower lip, prognathism, prominent chin, keeled chest, clinodactyly of the 5th finger, and hallux valgus. Furthermore, a general physical examination revealed seborrheic dermatitis on the face and scalp, as well as numerous nevi distributed over the entire skin surface.

Ophthalmological assessment showed bilateral corneal leukoma, nystagmus, and esotropia of the left eye. Neurological evaluation revealed dystonic movements of the hands, a wide-based ataxic gait, and impaired eye–hand coordination.

The electroencephalogram (EEG) showed no paroxysmal abnormalities. Ultrasound examination of the abdominal cavity, liver, kidneys, and heart revealed no significant abnormalities.

**Figure 1:**
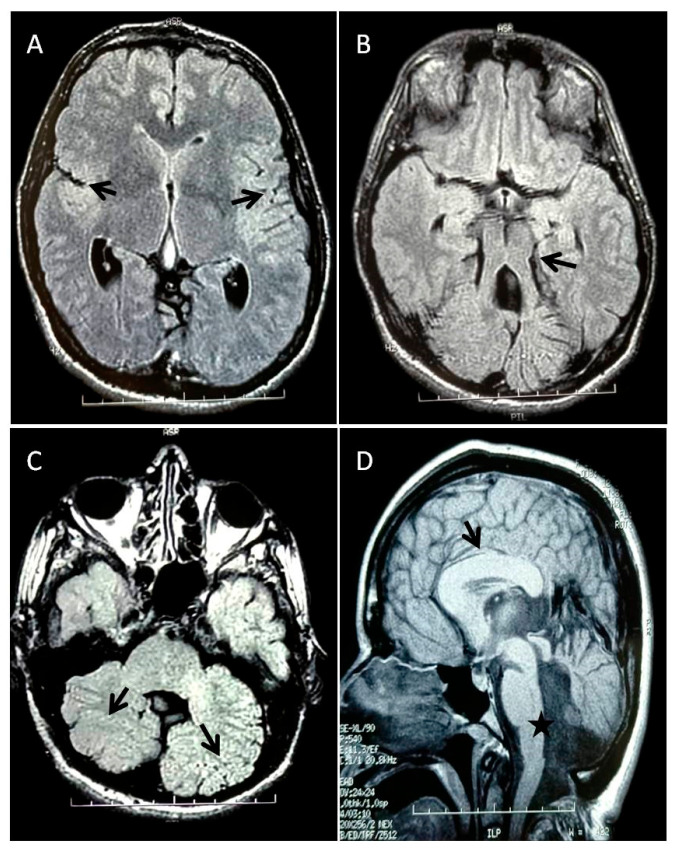
MRI brain images: T2 FLAIR axial images showing (**A**) reduced or absent opercularization (black arrows); (**B**) the molar tooth sign (black arrow) characterized by vermis hypoplasia, thickened cerebellar peduncles, and enlarged fourth ventricle; (**C**) diffusely abnormal foliation and fissuration of both cerebellar hemispheres (black arrows). FSE-XL sagittal image demonstrating (**D**) a thickened and stocky corpus callosum (black arrow) and an enlarged fourth ventricle communicating with a mega cisterna magna (black star).

Brain MRI performed at age 40 highlighted the molar tooth sign (MTS) in the posterior cranial fossa, characterized by cerebellar vermis hypoplasia, thickened cerebellar peduncles, dilation of the fourth ventricle communicating with a mega cisterna magna, and multiple cerebellar gyri malformations. In the supratentorial region, malformation of the Sylvian fissures due to reduced or absent opercularization was observed. Additionally, a thickened and stocky corpus callosum was detected (Figure 1A–D).

### 3.2. Genetic and Functional Analysis

**Figure 2:**
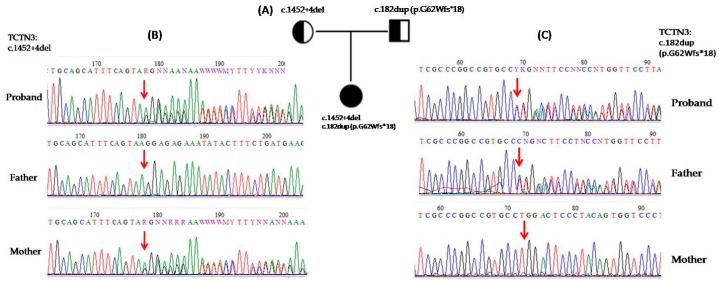
(**A**) Zygosity of the 49-year-old proband and her parents with two heterozygous variants in *TCTN3* (red arrow). (**B**,**C**) Sequencing chromatogram of the proband and her parents, showing c.182dup (p.G62Wfs*18) and c.1452+4del heterozygous variants in the *TCTN3* gene.

Whole-exome sequencing of the patient revealed c.182dup (p.G62Wfs*18) (rs763132585) and c.1452+4del (rs1555269741) variants in tectonic-3 (*TCTN3*) gene. The variants were confirmed by Sanger sequencing in the proband and her parents (Figure 2).

The first *TCTN3* variant, c.182dup (p.G62Wfs*18), was absent from the ExAC and 1000 Genomes Project databases, and it has a frequency of 0.0003 in gnomAD. It is considered pathogenic according to the variant interpretation guidelines of the American College of Medical Genetics (ACMG) (criteria PVS1, PM2, PP5) (Table 1), as supported by in silico predictions from MutationTaster and entries in the ClinVar database. This variant was inherited from the proband’s father.

The second *TCTN3* variant, c.1452+4del, was absent from the ExAC, 1000 Genomes Project, and gnomAD databases. According to the variant interpretation guidelines of the American College of Medical Genetics (ACMG) (criteria PP3, PM2) (Table 2), it is predicted to be disease-causing because it may alter splicing by weakening the donor splice site, as indicated by MutationTaster (wild-type score 0.99, mutant 0.25), SpliceRover (wild-type 0.55, mutant 0.00027), and SpliceAI (Donor Loss score 0.87) prediction software. However, ClinVar classifies this variant as of uncertain significance. This variant was inherited from the proband’s mother.

**Figure 3:**
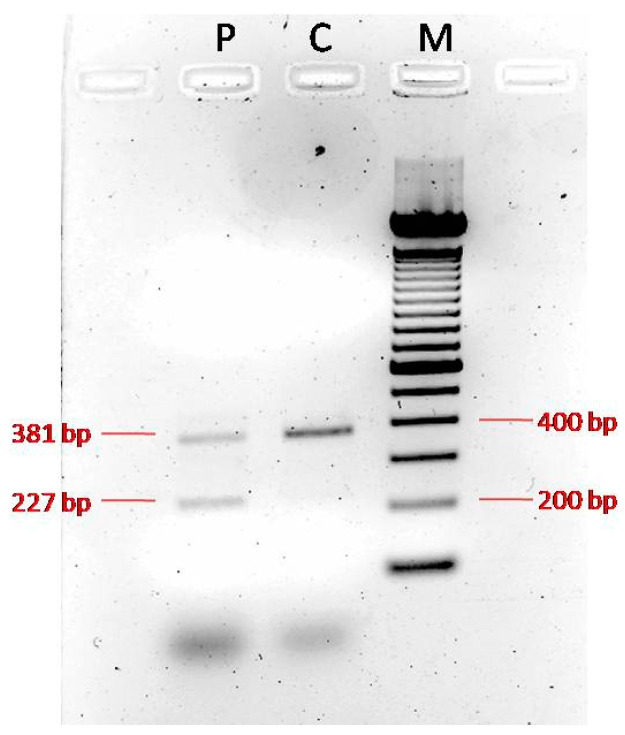
PCR of the cDNA derived from the proband showed two bands: a normal band of 381 bp and an aberrant band of 227 bp, confirming the skipping of exon 12 (Lane 1-P: Proband; Lane 2-C: Normal control; Lane 3-M: Marker).

To further support its pathogenicity, we analyzed the patient’s cDNA and confirmed that the variant the caused skipping of exon 12 (Figure 3, Figure S1), which likely resulted in the production of an abnormal protein.

## 4. Discussion

In this study, we report a European proband presenting with the molar tooth sign (MTS), thickened corpus callosum, ataxia, and intellectual disability (ID). Molecular analysis through whole-exome sequencing (WES) revealed two heterozygous variants in the *TCTN3* gene. Homozygous variants in *TCTN3* have been previously associated with Joubert syndrome type 18. Additionally, variants in this gene have been implicated in the etiology of Orofaciodigital syndrome type IV (OFD IV) and Meckel–Gruber syndrome (MKS)-like lethal phenotypes, where most variants are protein-truncating and linked to more severe clinical presentations. These severe phenotypes include brain anomalies, corpus callosum agenesis, vermian hypoplasia, the absence of the olfactory system, cystic kidney disease, severe skeletal dysplasia, facial dysmorphisms with a lobulated tongue, polydactyly affecting all four limbs, ductal plate proliferation in the liver, and occipital encephalocele [18,22,23].

Phenotypic variability associated with variants in the *TCTN3* gene can be explained by the type of variant identified. It is hypothesized that missense and splice site variants produce hypomorphic alleles, resulting in residual protein activity and consequently milder clinical features, such as Joubert syndrome [18,20]. Nonsense or frameshifting deletion variants may result in an absent or truncated protein, causing a more severe phenotype, as observed in OFD type IV and MKS-like lethal syndromes.

To date, 27 variants have been identified on the HGMD Professional database (18 disease-causing variants and 9 variants of uncertain significance). Previously, only five mutations, three missense mutations, one splicing, and one frameshift in the *TCTN3* gene have been identified as causative of JS [4,18,19,20,21]. Of these variants, Bachmann–Gagescu (2015) [4] reported the c.3G>A (p.M1I) homozygous pathogenic variant in one patient with coloboma. Thomas (2012) [18] reported two siblings of 13 and 6 years with the c.940G>A (p.G314R) homozygous variant, showing agenesis of the cerebellar vermis and MTS. Furthermore, this family exhibited additional features such as severe kyphoscoliosis in both affected siblings, camptodactyly and joint laxity in the older one, and a horseshoe kidney along with a ventricular septal defect in the younger. Ben-Salem (2014) [19] reported an unrelated family from the Middle East featuring JS with a digenic inheritance [(p.R479S) in the *TCTN3* gene and (p.I641N) in the AH1 gene]. Huppke (2015) [20], instead, reported a patient with the c.853-1G>T homozygous variant of the *TCTN3* gene, causing an alteration of the acceptor splice site, with the skipping of exon 7 and an in-frame deletion of 10 amino acids. The patient presented with agenesis of the cerebellar vermis and MTS, scoliosis, polydactyly, breathing abnormalities, ataxia, hypotonia, and ID. Finally, Huang (2022) [21], reported a fetus with the c.1441dupT (p.C481Lfs*133) homozygous variant, diagnosed with JS prenatally by MRI and ultrasound, which revealed the typical molar tooth sign (MTS), agenesis of the cerebellar vermis, polydactyly, malformation of cortical development, and dilation of the posterior fossa.

The variants identified in our patient, c.182dup (p.G62Wfs*18) and c.1452+4del, have not, to the best of our knowledge, been previously reported in individuals with *TCTN3*-related conditions. The first variant introduces a premature translational stop codon in the *TCTN3* gene and is expected to result in either an absent or truncated protein product and/or nonsense-mediated mRNA decay.

Regarding the second variant, since no functional studies have been performed, we investigated its impact on the transcript coding sequence. PCR amplification of the proband’s cDNA and that of a normal control produced distinct patterns. The normal control showed a single clear 381 bp wild-type band, whereas the proband’s cDNA yielded two bands, the normal 381 bp band and an aberrant 227 bp band, confirming skipping of exon 12 and suggesting the production of an abnormal protein. Unfortunately, sequencing of the isolated bands was not possible, preventing the validation of the transcript-level consequences. Both variants are located in exons 1 and the exon/intron boundary of exon 12, respectively, which correspond to the extracellular domain of the protein and are conserved across higher primates and rodents (http://www.uniprot.org/blast/). This conservation indicates that these variants are unlikely to be rare polymorphisms and probably underlie the phenotype observed in the patient.

The phenotype observed in our patient includes severe intellectual disability, ataxic gait, hypoplasia of the cerebellar vermis with the molar tooth sign (MTS), thickened corpus callosum, abnormal cerebellar hemispheres, dystonic movements, strabismus, and nystagmus. While agenesis of the corpus callosum (ACC) has been reported in approximately 3% of patients with JS [4], the thickening of the corpus callosum has not been previously described. Moreover, a thickened corpus callosum has never been reported in patients harboring pathogenic variants in the *TCTN3* gene. This novel finding may expand the phenotypic spectrum associated with *TCTN3*-related disorders.

Although the agenesis of the corpus callosum (ACC) is a common brain malformation associated with ID and neurodevelopmental disorders and linked to over 300 genes, the thickening of the corpus callosum is much rarer and has been associated with only a few genes. A thickened corpus callosum has been reported in individuals with de novo postzygotic or germline variants in genes such as *AKT3*, *PIK3R2*, *PIK3CA*, *HERC1*, and *MTOR*. These genes are involved in cell growth and proliferation and are molecular causes of Megalencephaly–Capillary Malformation Syndrome (MCAP), a rare genetic disorder characterized by primary megalencephaly, cutaneous vascular malformations, prenatal overgrowth, connective tissue dysplasia, digital anomalies, and body asymmetry.

Distinctive brain imaging features of MCAP include a thickened corpus callosum, polymicrogyria, lateral ventricles asymmetry, hydrocephalus, an enlarged cerebellum causing posterior fossa crowding, and cerebellar tonsillar herniation or ectopia [24,25]. The phenotype observed in this syndrome is caused by variants that disrupt the PI3K/Akt and mTOR signaling pathways. The PI3K/AKT/mTOR pathway is responsible for controlling important cellular responses like cell growth and proliferation, survival, migration, and metabolism [26].

TCTN3 belongs to the tectonic family of proteins, which include TCTN1, TCTN2, and TCTN3, a group of proteins existing in the cilium transition zone (TZ). TCTN proteins are considered to play a vital role in trafficking proteins into the cilia and are required for ciliary development and ciliogenesis. Furthermore, they play a key role in many signaling pathways, including Shh, crucial for development and tissue homeostasis [27]. The Shh pathway influences cellular fate by controlling the PI3K/Akt and mTOR signaling pathways. It could be hypothesized that variants of the *TCTN3* gene cause the deregulation of the Shh pathway and indirectly also of the PI3K/AKT/mTOR pathway. In fact, studies on mutated mice demonstrated that Tctn3^KO−/−^ results in the abnormal expression of Shh signaling pathway-related genes and also cause abnormal neural tube patterning and neuronal apoptosis [17].

This work confirms existing data suggesting that variants in the *TCTN3* gene are associated with a broad clinical spectrum, ranging from mild to lethal phenotypes [28]. Indeed, our patient presents a thickened corpus callosum but does not exhibit renal or cardiac anomalies, further supporting the phenotypic heterogeneity linked to *TCTN3* variants.

In summary, we report an additional patient with clinical signs of Joubert syndrome carrying two novel variants in the *TCTN3* gene and exhibiting a thickened corpus callosum. This finding expands the phenotype–genotype spectrum associated with *TCTN3*, underscoring the importance of considering a wide variability of clinical signs when suspecting a *TCTN3*-associated syndrome, given the gene’s crucial role in cellular differentiation, migration, and neuronal proliferation

## Figures and Tables

**Table 1:** Variant classification according to the ACMG criteria.

c.182dup (p.G62Wfs*18)		
Criteria for Classifying Variants	Category Code	Description
Pathogenic Very Strong:	PVS1	Null variant in a gene where loss of function is a known mechanism of disease
Pathogenic Moderate:	PM2	Extremely low frequency in gnomAD population databases
Pathogenic Supporting:	PP5	A reputable source recently reported the variant as pathogenic, but the evidence is not available to the laboratory to perform an independent evaluation
ACMG variant classification		Pathogenic

**Table 2:** Variant classification according to the ACMG criteria.

c.1452+4del		
Criteria for Classifying Variants	Category Code	Description
Pathogenic Moderate:	PP3	For a missense or a splicing region variant, computational prediction tools unanimously support a deleterious effect on the gene
Pathogenic Moderate:	PM2	Extremely low frequency in gnomAD population databases
ACMG variant classification		Uncertain significance

## Data Availability

Data are available from the corresponding author upon a reasonable request.

## Supplementary Materials

The following supporting information can be downloaded at https://www.mdpi.com/article/10.3390/genes16060706/s1, Figure S1: cDNA PCR GEL.

## Abbreviations

The following abbreviations are used in this manuscript:

JS	Joubert syndrome
MTS	Molar tooth sign
MRI	Magnetic resonance imaging
CNS	Central nervous system
MKS	Meckel–Gruber Syndrome
OFD	Orofaciodigital syndrome IV
ID	Intellectual disability
WES	Whole-exome sequencing
EEG	Electroencephalogram
ACC	Agenesis of the corpus callosum
MCAP	Megalencephaly-Capillary Malformation Syndrome
