# Supplementary figures and images for: Expansion of the Genotypic and Phenotypic Spectrum of TCTN3-Related Joubert Syndrome
Source: Genes (Basel). 2025 Jun 13;16(6):706. doi: 10.3390/genes16060706 (PMC12193267; doi:10.3390/genes16060706)

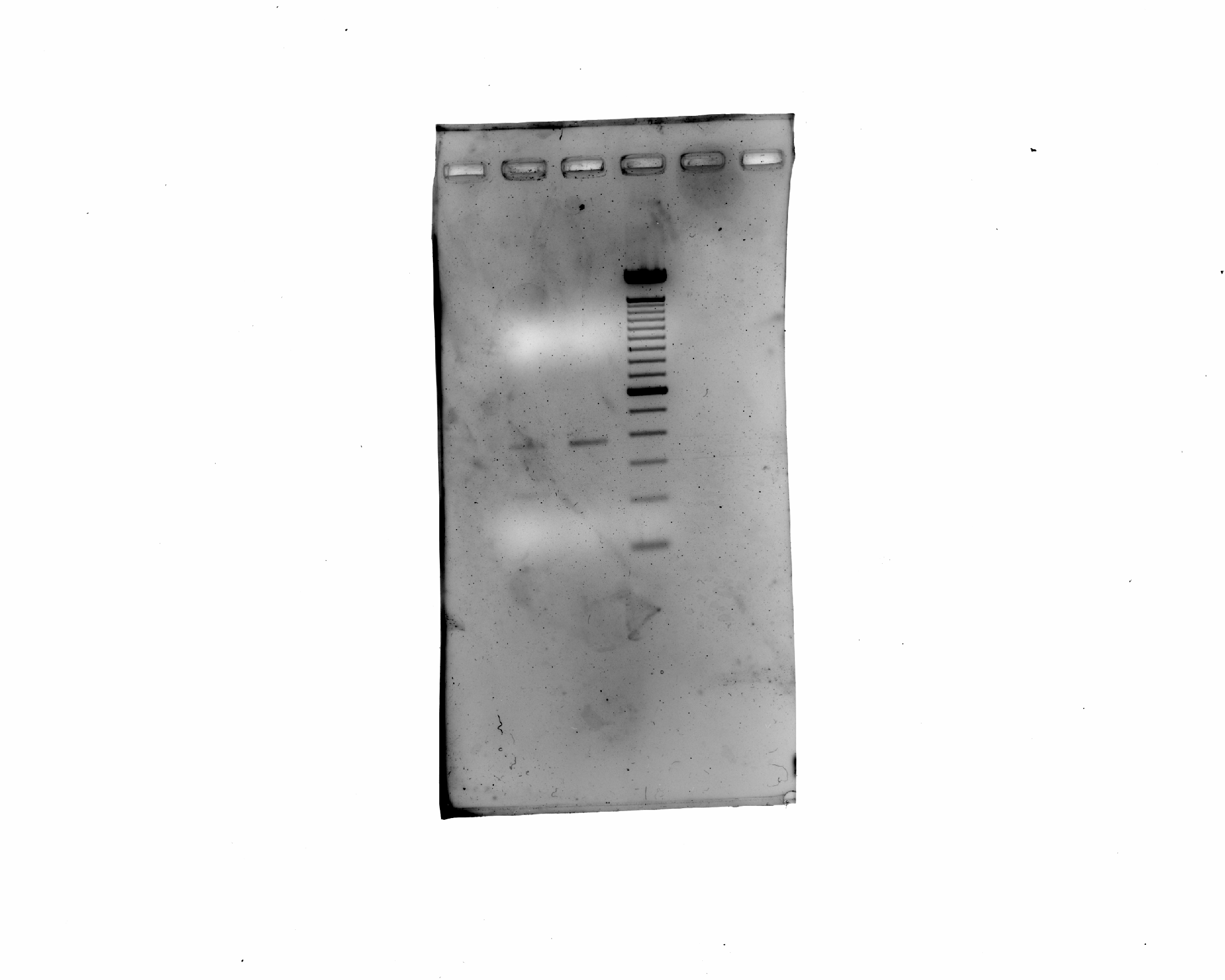

Supplement: Supplementary file 1 [file genes-16-00706-s001.zip › cDNA PCR GEL.tif]
